# Environmental Influences on Pigeonpea-*Fusarium udum* Interactions and Stability of Genotypes to Fusarium Wilt

**DOI:** 10.3389/fpls.2016.00253

**Published:** 2016-03-07

**Authors:** Mamta Sharma, Raju Ghosh, Rameshwar Telangre, Abhishek Rathore, Muhammad Saifulla, Dayananda M. Mahalinga, Deep R. Saxena, Yogendra K. Jain

**Affiliations:** ^1^Research Programme Grain Legumes, International Crop Research Institute for the Semi-Arid TropicsTelangana, India; ^2^Department of Plant Pathology, University of Agricultural Sciences, GKVKKarnataka, India; ^3^Department of Plant Pathology, Agricultural Research StationKarnataka, India; ^4^Department of Plant Pathology, R A K College of AgricultureMadhya Pradesh, India; ^5^Department of Plant Pathology, Zonal Agricultural Research StationMadhya Pradesh, India

**Keywords:** fusarium wilt, GGE biplot, genetic diversity, host plant resistance, multi-environment, pigeonpea

## Abstract

Fusarium wilt (*Fusarium udum* Butler) is an important biotic constraint to pigeonpea (*Cajanus cajan* L.) production worldwide. Breeding for fusarium wilt resistance continues to be an integral part of genetic improvement of pigeonpea. Therefore, the study was aimed at identifying and validating resistant genotypes to fusarium wilt and determining the magnitude of genotype × environment (G × E) interactions through multi-environment and multi-year screening. A total of 976 genotypes including germplasm and breeding lines were screened against wilt using wilt sick plot at Patancheru, India. Ninety two genotypes resistant to wilt were tested for a further two years using wilt sick plot at Patancheru. A Pigeonpea Wilt Nursery (PWN) comprising of 29 genotypes was then established. PWN was evaluated at nine locations representing different agro-climatic zones of India for wilt resistance during two crop seasons 2007/08 and 2008/09. Genotypes (G), environment (E), and G × E interactions were examined by biplot which partitioned the main effect into G, E, and G × E interactions with significant levels (*p* ≤ 0.001) being obtained for wilt incidence. The genotype contributed 36.51% of resistance variation followed by the environment (29.32%). A GGE biplot integrated with a boxplot and multiple comparison tests enabled us to identify seven stable genotypes (ICPL 20109, ICPL 20096, ICPL 20115, ICPL 20116, ICPL 20102, ICPL 20106, and ICPL 20094) based on their performance across diverse environments. These genotypes have broad based resistance and can be exploited in pigeonpea breeding programs.

## Introduction

Pigeonpea (*Cajanus cajan* L. Millisp.) commonly known as redgram, is a low input, rain fed crop with characteristics that provide economic returns from each and every part of the plant (Saxena, 2006). Pigeonpea cultivation has a direct bearing on the overall economic and financial well-being and the nutritional status of the subsistence farmers in the subcontinent of Eastern and Southern Africa, Asia, and South America (Hillocks et al., 2000; Souframanien et al., 2003). It also restores soil fertility by fixing atmospheric nitrogen and has the ability to solubilize fixed phosphorus (Ae et al., 1990). India is the principal pigeonpea growing country and contributes nearly 90% of world's acreage and production, followed by Myanmar, Kenya, and Malawi (FAOSTAT, 2013).

**Figure 1:**
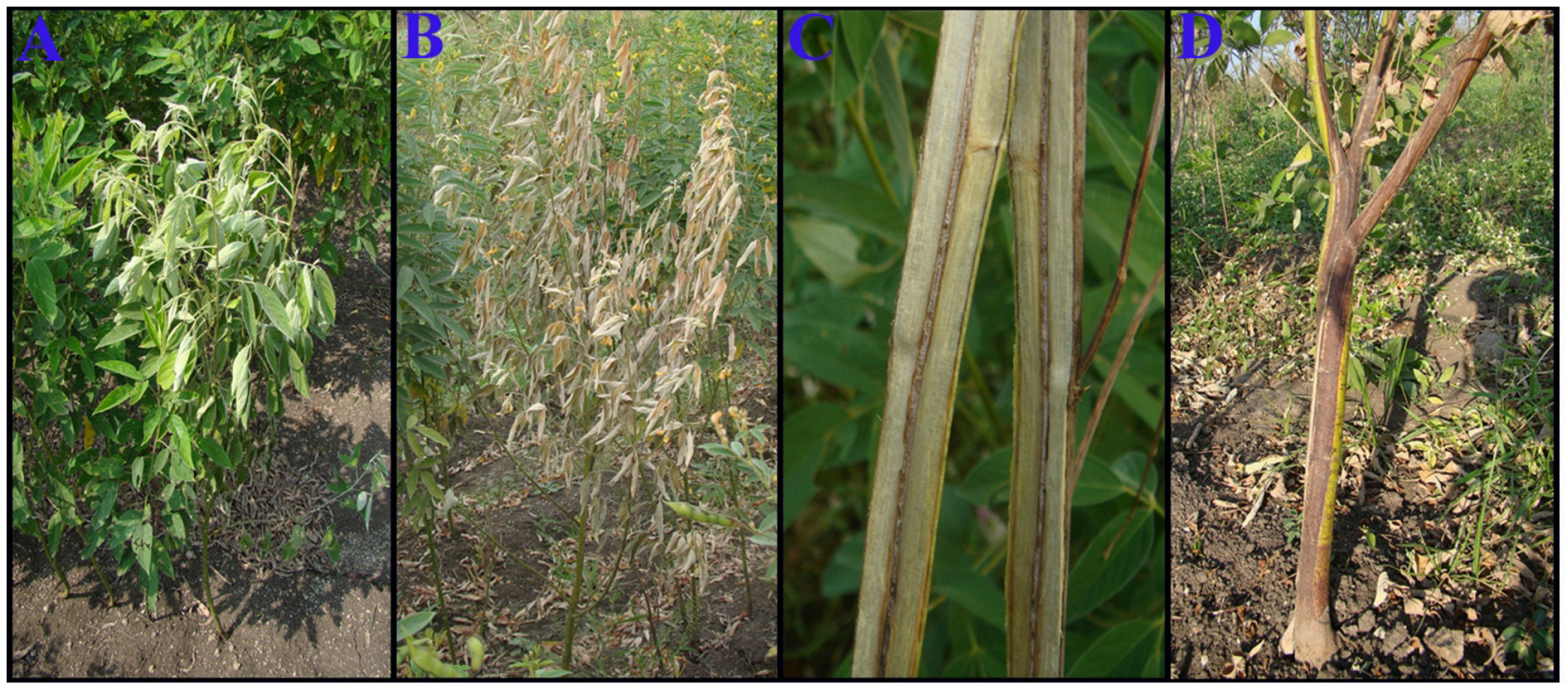
**Symptoms of Fusarium wilt on infected pigeonpea. (A)** Loss of turgidity. **(B)** Slight inter-veinal chlorosis and drying of leaves. **(C)** Internal browning of xylem vessels. **(D)** Purple band on stem extending upwards from the base.

Wilt caused by *Fusarium udum* Butler, is the major constraint to pigeonpea production worldwide (Kannaiyan et al., 1984). The incidence of disease has been reported from 30 to 60% at flowering and crop maturity stages (Kannaiyan and Nene, 1981), however it can also cause yield losses up to 100% in susceptible cultivars (Kannaiyan et al., 1984; Reddy et al., 1990; Okiror, 1999; Dhar et al., 2005). Wilt can be diagnosed by symptoms like loss of turgidity, slight inter-veinal chlorosis, internal browning of xylem vessels, and a purple band on stem extending upwards from the base (Figure 1). An updated review of biology, pathogenicity, epidemiology, and management of pigeonpea wilt has been recently published by Pande et al. (2013a).

Management of wilt is essential to ensure stable pigeonpea production. Fungicide is not economical and doesn't give complete protection. Hence it is imperative to identify stable sources of resistance and exploit them to develop resistant varieties of pigeonpea through breeding approaches. A number of strategies to deal with wilt were identified over the past two decades by screening pigeonpea genotypes under national and international programs. Some of these sources have been effectively used in breeding programs and released as resistant varieties for disease prone areas (Nene and Kannaiyan, 1982; Okiror, 1999; Sharma and Pande, 2011; Sharma et al., 2012a).

The existence of variants/races of *F. udum* is a major drawback for breeding programs for wilt resistance (Mishra and Dhar, 2003; Mishra, 2004; Singh et al., 2011; Tiwari and Dhar, 2011). *F. udum* isolates from diverse geographical origins have been shown to exhibit high variability in their virulence (Mishra and Dhar, 2003; Mamta Sharma, unpublished data). This reinforces the need to search for additional stable sources of resistance to wilt. Multi-location and multi-year testing of genotypes is essential to check stability of genotype resistance across pigeonpea growing regions. Genotype and genotype × environment interaction (GGE) Biplot analysis results can discriminate between expected and realized responses of genotypes and has been widely used in recent years to determine the stability of disease resistance through multi-location trials. GGE biplot is an effective method based on principal component analysis (PCA) to fully explore data. It allows visual examination of the relationships among the test environments, genotypes, and the GE interactions. It is an effective tool for: (i) mega-environment analysis, whereby specific genotypes can be recommended for specific mega-environments (Yan and Kang, 2003; Yan and Tinker, 2006), (ii) genotype evaluation (the mean performance and stability), and (iii) environmental evaluation (the power to discriminate among genotypes in target environments; Ding et al., 2007). The interaction was primarily reported to evaluate the yield and other traits in multi-location trials (Yan et al., 2000; Yan and Kang, 2003). Recently this methodology has been used to characterize and identify stability of germplasm, breeding lines and cultivars resistance to diseases such as Fusarium head blight and powdery mildew in wheat (Kadariya et al., 2008; Lillemo et al., 2010), ascochyta blight in faba bean (Rubiales et al., 2012), Fusarium wilt and ascochyta blight in chickpea (Sharma et al., 2012b; Pande et al., 2013b), and sterility mosaic disease in pigeonpea (Sharma et al., 2015). Therefore, the present study was conducted with an objective to identify and validate pigeonpea genotypes resistant to *F. udum* through multi-environment and multi-year evaluations and identify stability of their resistance.

## Materials and methods

### Plant material

A total of 976 genotypes obtained from the pigeonpea breeding program (ICRISAT, Patancheru) were evaluated for wilt using wilt sick plot during 2004/05 crop season. Resistant lines (≤10 % wilt incidence) selected after preliminary screening were further evaluated for 2 more years by wilt sick plot at ICRISAT (Patancheru) during 2005/06 and 2006/07 crop seasons. Finally a Pigeonpea Wilt Nursery (PWN) of 29 genotypes was constituted from the above three subsequent evaluations based on their total wilt reaction (≤10% wilt incidence) for multi-year and multi-environment screening. The PWN consisted of 4 germplasm accessions, 24 breeding lines, and a highly susceptible commercial check with days to maturity ranging from 150 to 252 days (Table 1). Additionally one local susceptible check from the test location was included to ensure disease pressure. The summary of the pedigrees for the 29 genotypes used in this study are presented in Table 1.

**Table 1:** **Pedigrees and agronomic traits of the pigeonpea genotypes used in the Pigeonpea Wilt Nursery during 2007/08 and 2008/09**.

**Serial No**.	**Genotype**	**Type**	**Pedigree**	**Days to 50% flowering**	**Days to 50% maturity**
1	ICP 9174	Gene bank accession	ICRISAT-COOP-N/A	161	252
2	ICP 12749	Gene bank accession	ICP 7065 × 7035-F4B-S218X	138	218
3	ICP 14819	Gene bank accession	ICRISAT-COOP-0624	158	210
4	ICPL 20093	Breeding line	ICPX 900148-7^*^	127	183
5	ICPL 20094	Breeding line	ICPX 900152-^*^	129	185
6	ICPL 20096	Breeding line	ICPX 900146-^*^	127	185
7	ICPL 20097	Breeding line	ICPX 900146-^*^	131	187
8	ICPL 20098	Breeding line	ICPX 900146-^*^	128	184
9	ICPL 20099	Breeding line	ICPX 900155-^*^	127	184
10	ICPL 20100	Breeding line	ICPX 900148-^*^	127	183
11	ICPL 20101	Breeding line	ICPX 900147-^*^	128	185
12	ICPL 20102	Breeding line	ICPX 900148-9^*^	126	181
13	ICPL 20103	Breeding line	ICPX 900150-^*^	131	186
14	ICPL 20106	Breeding line	IPH487 Inbred-12^*^	127	182
15	ICPL 20107	Breeding line	IPH487 Inbred-2^*^	130	185
16	ICPL 20109	Breeding line	IPH487 Inbred-9^*^	131	187
17	ICPL 20110	Breeding line	IPH487 Inbred-7^*^	130	186
18	ICPL 20113	Breeding line	IPH487 Inbred-1^*^	129	185
19	ICPL 20114	Breeding line	IPH487 Inbred-11^*^	129	184
20	ICPL 20115	Breeding line	IPH487 Inbred-14^*^	125	181
21	ICPL 20116	Breeding line	IPH487 Inbred-4^*^	125	181
22	ICPL 20120	Breeding line	IPH487 Inbred-17^*^	131	186
23	ICPL 20126	Breeding line	GUPH 1126 Inbred-3^*^	128	183
24	ICPL 20128	Breeding line	GUPH 1126 Inbred-11^*^	126	182
25	ICPL 20129	Breeding line	GUPH 1126 Inbred-10^*^	131	185
26	ICPL 20132	Breeding line	GUPH 1126 Inbred-1^*^	129	184
27	ICPL 20134	Breeding line	GUPH 1126 Inbred-7^*^	129	183
28	KPBR 80-2-4	Accession	Gene bank accession	165	215
29	ICP 2376^**^	Accession	ICRISAT-COOP-0436	110	150
30	Local wilt sus. check	–	–	–	–

* *Selfed population*,

** *Susceptible check*.

### Test locations

**Figure 2:**
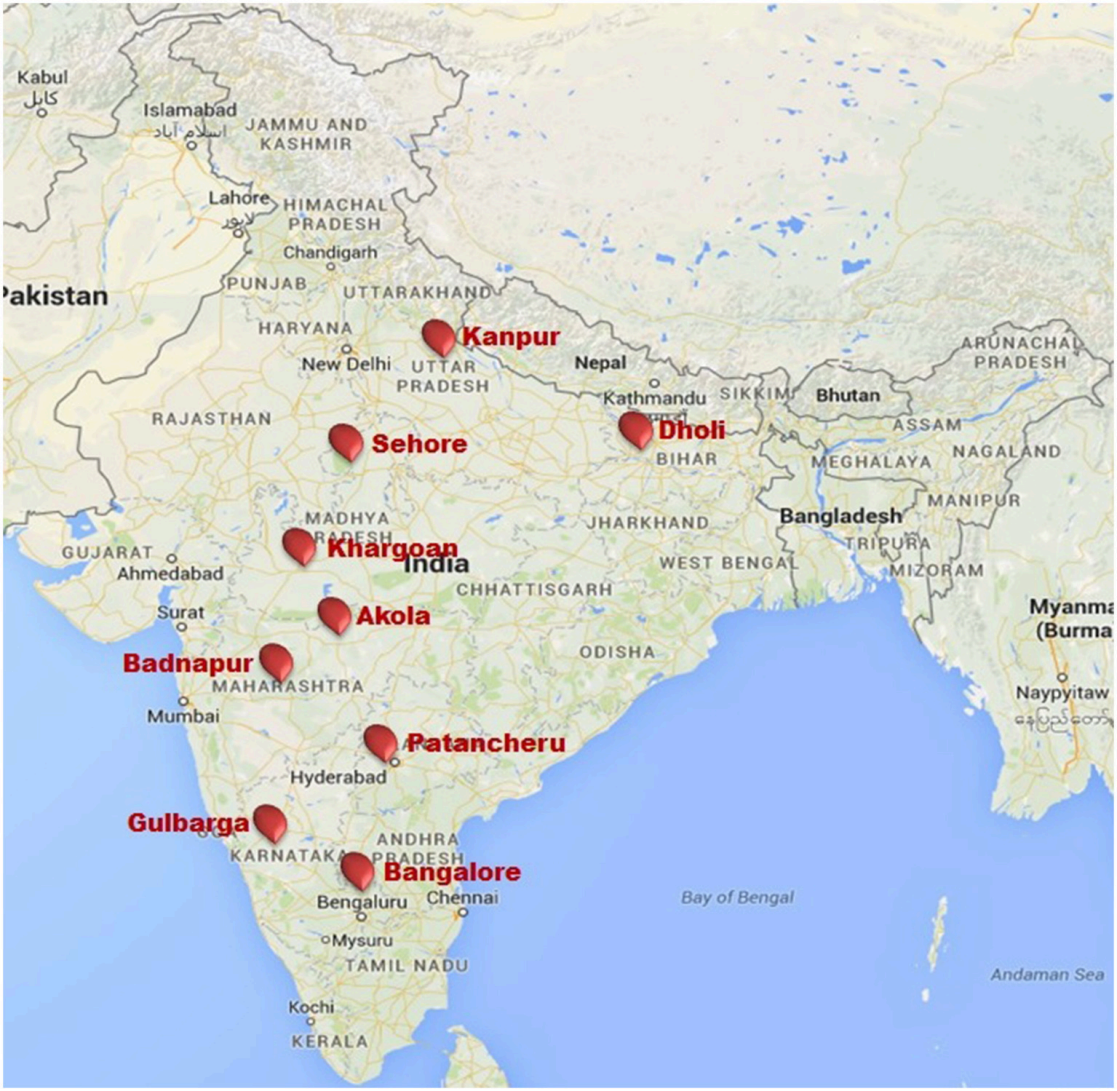
**Pigeonpea wilt nursery testing locations in India during 2007/08 and 2008/09**.

The PWN was evaluated against wilt at nine locations in India (Akola, Badnapur, Bangalore Dholi, Gulbarga, Kanpur, Khargoan, Patancheru, and Sehore). Test locations were selected based on different agro-climatic zones (Table 2) and availability of wilt sick plot. These sites represented six major pigeonpea producing states (Andhra Pradesh, Bihar, Karnataka, Madhya Pradesh, Maharashtra, and Uttar Pradesh) with a wide diversity in latitude from 12°58′ at Bangalore to 26°26′ at Kanpur, longitude from 75°43′ at Badnapur to 85°35′ at Dholi and altitude ranging 52.2 m of Dholi to 920 m of Bangalore from mean sea level. Details of the tested environments (location, state, latitude, longitude, altitude and their agro-climatic zone) are given in Table 2 and also indicated in map (Figure 2).

**Table 2:** **Details of test environments used for evaluation of pigeonpea genotypes against wilt disease**.

**Location**	**State**	Environments^*^	**Latitude (N)**	**Longitude (E)**	**Altitude (m)**	Agro-climatic zone^**^	**Soil type**	**Annual rainfall (mm)**
Akola	Maharashtra	Ak-07	20°42′	76°59′	282	PZ	Vertisol	915.2
		Ak-08						593.4
Badnapur	Maharashtra	Bd-07	19°23′	75°43′	582	PZ	Vertisol	485.2
		Bd-08						113.8
Bangalore	Karnataka	Bn-07	12°58′	77°35′	920	SZ	Alfisol	804.8
		Bn-08						884.6
Gulbarga	Karnataka	Gu-07	17°19′	76°50′	454	SZ	Vertisol	764.2
		Gu-08						744.0
Patancheru	Andhra Pradesh	Pa-07	17°31′	78°15′	545	SZ	Vertisol	707.0
		Pa-08						1105.0
Dholi	Bihar	Dh-07	25°59′	85°35′	52.2	NEPZ	Alfisol	2624.8
		Dh-08						1830.3
Kanpur	Uttar Pradesh	Ka-07	26°26′	80°19′	126	NEPZ	Alfisol	542.6
		Ka-08						687.1
Khargone	Madhya Pradesh	Kh-07	21°49′	75°36′	252	CZ	Vertisol	995.5
		Kh-08						472.9
Sehore	Madhya Pradesh	Se-07	23°11′	77°04′	457	CZ	Vertisol	893.0
		Se-08						679.5

* *Environment is denoted as first two letters of each locations followed by year of screening*.

** *PZ, Plateau zone; SZ, South zone; NEPZ, North eastern plane zone; CZ, Central zone*.

### Multi-environment evaluation and validation

**Figure 3:**
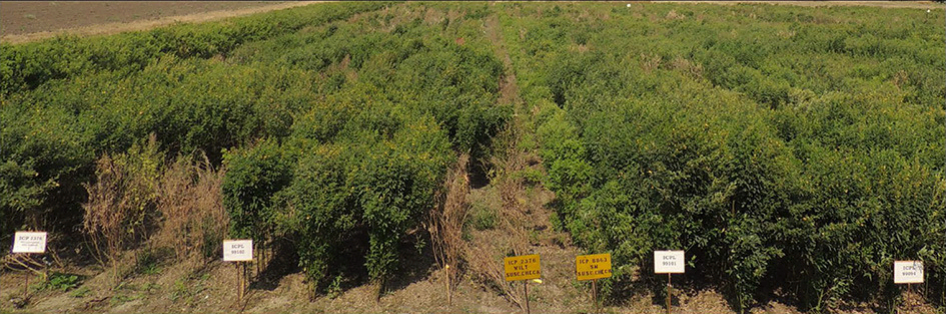
**Field screening (wilt sick plot) for Fusarium wilt disease of pigeonpea**.

The PWN constituting of 29 genotypes with consistent and higher levels of resistance were evaluated at multi-environments in 2007/2008 and 2008/2009. Seed stocks of test genotypes (genetically pure) were increased and maintained at ICRISAT (Patancheru) and sub-sampled to supply to collaborators at key locations for wilt screening in the major pigeonpea growing areas. To ensure the genetic integrity, seeds of tested genotypes were maintained by selfing. The nursery was laid out in a randomized complete block design (RCBD) with two replicates. Each genotype was grown in one row, 4 m in length with row-to-row spacing of 75 cm and plant-to-plant spacing of 10 cm within the row. A local susceptible check was planted at every 5th row (Figure 3).

### Data collection and analysis

Data on wilt incidence (%) was recorded at seedling, flowering and maturity stage of the crop at each location. Cumulative incidence of all the three stages was calculated using the following formula:

$$\%\text{ disease incidence}=\;\frac{\text{No}.\text{ of infected plants}}{\text{Total no}.\text{ of plants}}\times \text{100}$$

Depending upon the range of wilt incidence, the test entries were categorized as resistant (<10.0% incidence), moderately resistant (10.1–20.0% incidence), susceptible (20.1–40.0% incidence), and highly susceptible (>40% incidence). Prior to analysis, the percentage data was arcsine transformed to make residual normal (Gomez and Gomez, 1984).

The transformed and replicated data was subjected to an analysis of variance (ANOVA) to know the level of significance of the genotypes, environment and their interaction for individual year as well as for combined years using GenStat software (17th edition). For the analysis, genotype (G) and environment (E) were considered as a fixed effect. Since the local susceptible check line was different for each location it was eliminated from the analysis. However, the susceptible check (ICP 2376) common at all locations was kept to compare disease reaction. Significance of mean differences within genotypes and environments was tested by the Student's *t*-test in combination with Bonferroni correction at the *P* = 0.05 level of probability. Also, a Boxplot (environment × incidence and genotype × incidence) was generated to visualize the distribution pattern of disease incidence among 29 genotypes across environments (Wiik and Rosenqvist, 2010). To identify the relationship between environments, Spearman's correlation was calculated by comparing the disease incidence of genotypes and hierarchical cluster of environments, which was generated using Euclidean similarity coefficient.

To determine resistance stability of genotypes across environments, GGE biplot analyses was conducted. GGE biplot is a method of geographical analysis of multi-environment data (Yan, 2001; Yan and Falk, 2002). Eighteen environments (Table 2) and 29 genotypes (Table 1) were used in this analysis, including one common susceptible check. It displays the main genotype effect (G) and the genotype × environment (G × E) interaction of multi-environment tests. GGE model used to determine the stability of genotypes across environments is:

$$\begin{array}{l} Yij-\;\mu -\;\beta j=\overset{k}{\underset{i=1}{\sum }}\lambda l\xi il\eta lj+\;\varepsilon ij \end{array}$$

where *Yij* is the mean genotype incidence *i* in environment *j*, μ is the grand mean, β*j* is the environment *j* main effect, *n* is the singular value, λ and ξ are the singular vectors for genotype and environment for *n* = 1, 2,…, respectively, and ε*ij* is the residual effect. This biplot was constructed by plotting the first two principal components (PC1 and PC2) derived from single value decomposition of the environment centered data. Genotype and environments were displayed in the same plot. Each genotype and environment was defined by their respective scores on the two PCs. Angles between the environment vectors were used to judge the correlation among the environments (Yan and Kang, 2003). The length of vector represents the genotypic variability in the respective environment. In order to assay the stability of genotypes, the average environment coordinate (AEC) is plotted by taking the mean of PC1 and PC2 scores for environments. A performance line passing through the origin of the biplot used to determine the mean performance of the genotype. The arrow on the performance line represents an increase in mean disease incidence i.e., higher susceptibility (Sharma et al., 2012b).

## Results

### Preliminary screening for identification of resistant genotypes

Preliminary screening of the 976 genotypes revealed a broad range of response to Fusarium wilt in wilt sick plot during 2004/05 at ICRISAT (Patancheru) and allowed the removal of susceptible materials and the selection of 92 resistant genotypes for further screening. These 92 genotypes were further screened for two consecutive years (2005/06 to 2006/07) by wilt sick plot and finally 28 highly resistant genotypes with consistent disease incidence ≤10% were chosen for the creation of the PWN.

### Response of genotypes to wilt

The pooled ANOVA showed that G, E, and G × E effects were significant (Table 3). Disease incidence was affected by the genotype across the environments as genotype accounted for 36.51% of the variance. G × E and E accounted for 33.82 and 29.32% respectively, indicating the confounding influence of the environment on evaluation in different locations. Significant differences were found in the genotype and year × genotype effect for wilt incidence at all the locations (Table 4).

**Table 3:** **Analysis of variance for wilt per cent incidence of 29 pigeonpea genotypes evaluated at nine locations under artificial epiphytotic conditions during 2007/08 and 2008/09**.

**Source of variation**	**Degree of freedom**	**Sum of square**	**Mean sum of square**	***P***	Variation (%)^*^
Genotype (G)	28	75749.31	2705.33	<0.001	36.51
Environment (E)	17	60838.63	3578.74	<0.001	29.32
G × E	476	70178.37	147.43	<0.001	33.82

* *Relative percentage contribution of each source of variation to the total variance*.

**Table 4:** **Location wise combined analysis of variance of F statistic value for wilt incidence of 29 genotypes during 2007/08 and 2008/09**.

**Source of variation**	**Locations**								
	**Akola**	**Badnapur**	**Bangalore**	**Dholi**	**Gulbarga**	**Kanpur**	**Khargoan**	**Patancheru**	**Sehore**
Degrees of Freedom	58	58	58	58	58	58	58	58	58
Year (Y)	2.48	38.13^**^	43.84^**^	129.2^**^	0.84	4773.26^**^	14.67^**^	49.06^**^	7749.74^**^
Genotype (G)	436.44^**^	380.19^**^	804.61^**^	211.32^**^	172.45^**^	779.18^**^	611.44^**^	331.73^**^	2970.71^**^
Y × G	38.54^**^	78.32^**^	164.94^**^	38.24^**^	10.37^**^	82.28^**^	339.45^**^	25.39^**^	323.88^**^

** *Significant at P = 0.01*.

**Figure 4:**
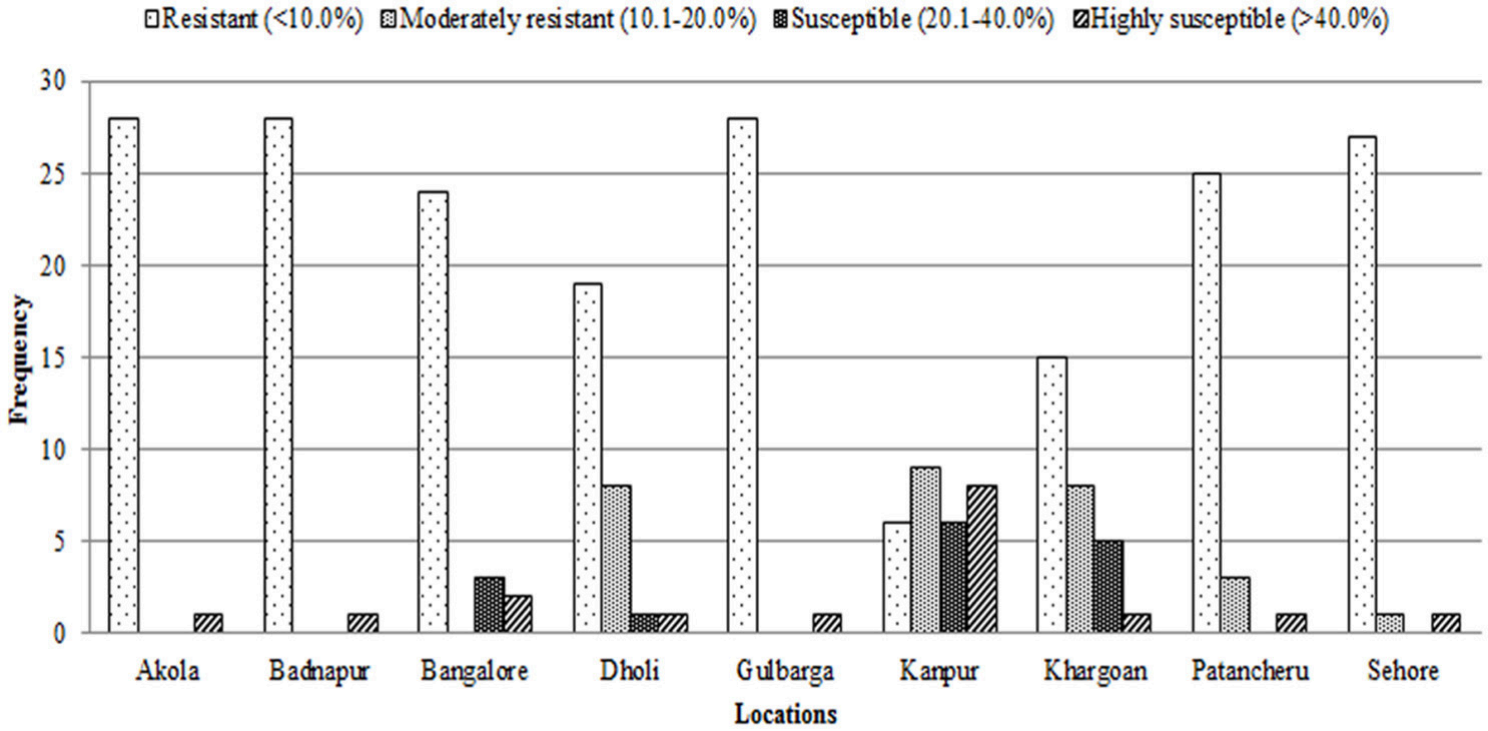
**Frequency distribution of 29 pigeonpea genotypes for levels of Fusarium wilt disease at nine locations in India over 2 years (2007/2008 and 2008/2009)**. Rating of genotype reaction: resistant = 0–10% wilt incidence; moderately resistant = 10.1–20% wilt incidence; susceptible = 20.1–40% and highly susceptible = 40.1–100%.

**Figure 5:**
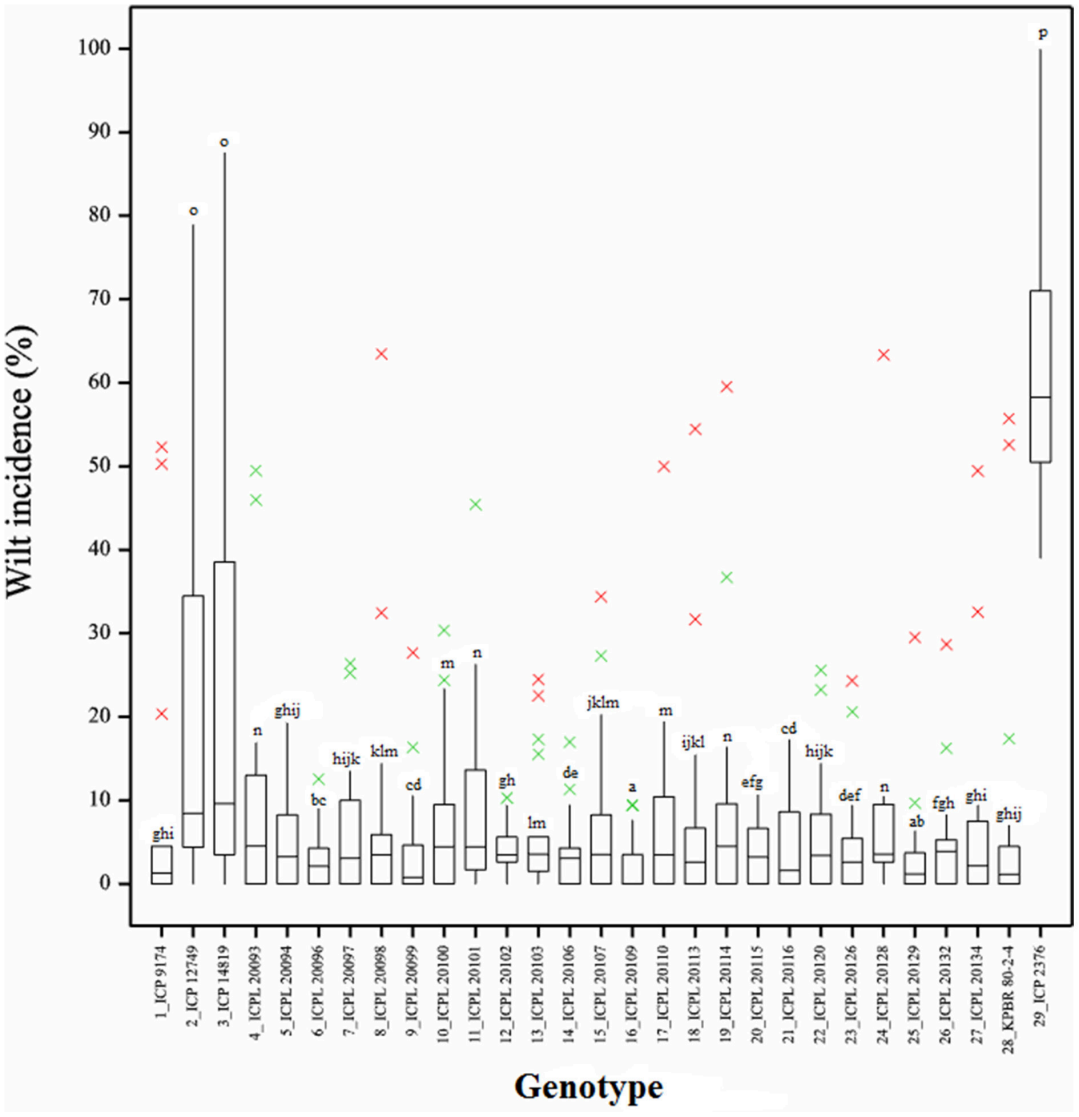
**Boxplot showing the differences in percent wilt incidence of each genotype across 18 environments**. Box edges represent the upper and lower quantile with median value shown in the middle of the box. Whiskers represented by green “×” symbol. Individuals falling outside the range of whiskers shown as red “×” symbol.

**Figure 6:**
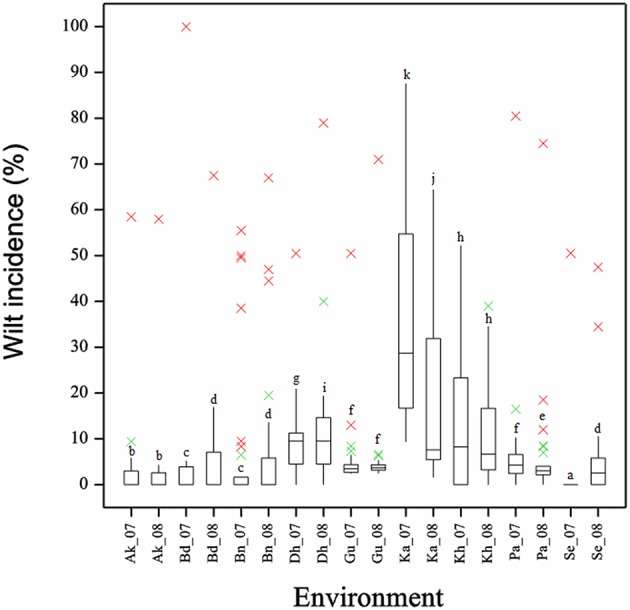
**Boxplot showing the differences in per cent wilt incidence for 18 environments across 29 genotypes**. Box edges represent the upper and lower quantile with median value shown in the middle of the box. Whiskers represented by green “×” symbol. Genotypes falling outside the range of whiskers shown as red “×” symbol.

**Figure 7:**
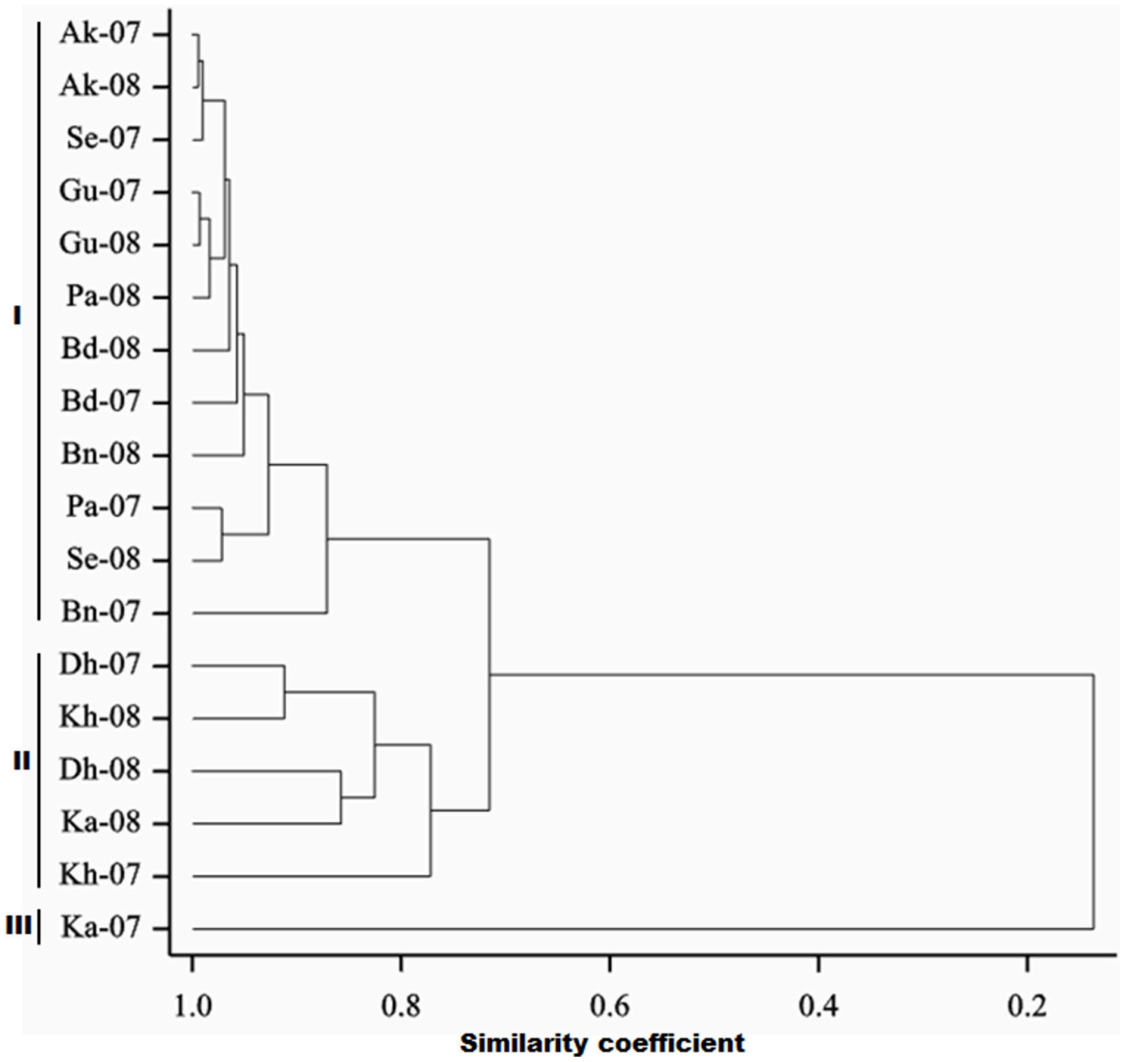
**Hierarchical cluster analysis showing the relationship between 18 environments**.

Frequency distribution of combined wilt incidence for both years is outlined in Figure 4 and the range of incidence of genotype across environments is represented by box plot (Figure 5). The variability in wilt incidence with respect to genotypes was more at the Kanpur location, as shown by a frequency distribution of disease incidence of different genotypes (Figure 4 and Table 5). Ka-07 had the highest (36.15%) as well as widest range of wilt incidence (6.4–73.7) across genotypes while Se-07 had the lowest (1.56%) wilt incidence across genotypes (Figure 6). Mean wilt incidence at the nine locations varied from 3.2 to 27.2% (Table 5). Akola had a low mean wilt incidence (3.2%) followed by Sehore (3.6%) and Badnapur (5.5%). Conversely, Kanpur recorded the highest mean wilt incidence (27.2%) followed by Khargone (11.8%) and Dholi (10.7%). Mean performance of all the genotypes was less than 10.0%, except for ICP 12749 and ICP 14819 where the mean disease incidence was 20.5 and 19.6%, respectively (Table 5). The genotypes ICPL 20109, ICPL 20096, ICPL 20115, and ICPL 20102 showed a greater degree of resistance with the wilt incidence 2.6, 2.8, 4.0, and 4.1% respectively across 18 environments. Further, all 28 genotypes were found to have resistance to wilt at three locations (Akola, Badnapur and Gulbarga), 27 at Sehore, 24 each at Bangalore and Patancheru, 18 at Dholi, 15 at Khargone, and 6 at Kanpur (Figure 4 and Table 5). Correlation between environments showed that environments, for instance Bd-08 and Pa-08, had a significant positive correlation (*r* = 0.76; Supplementary Table 1) indicating that the environments were closely related to each other with respect to disease incidence. Finally, the relationship between environments is shown by hierarchical clustering (Figure 7). All the environments were found to be grouped in three major clusters with 12 environments in cluster I, five in cluster II, and only one in cluster I. Cluster III placed separately as is evidenced from its highly virulent reaction to pigeonpea genotypes.

**Table 5:** **Mean wilt incidence (%) of pigeonpea genotypes across nine locations during 2007/08 and 2008/09**.

**Sr. No**.	**Entry**	**Akola**	**Badnapur**	**Bangalore**	**Dholi**	**Gulbarga**	**Kanpur**	**Khargoan**	**Patancheru**	**Sehore**	**Mean^a^**	**BMC**
1	ICP 9174	1.5	0.0	0.0	4.5	3.1	35.4	26.2	0.0	1.6	10.1	ghi
2	ICP 12749	4.3	5.1	26.8	9.8	4.4	69.8	33.1	14.4	17.2	23.5	o
3	ICP 14819	3.4	8.6	41.5	25.8	4.8	73.7	3.3	10.3	5.3	22.8	o
4	ICPL 20093	0.0	8.5	24.8	7.8	8.3	25.7	8.6	2.7	2.8	13.7	n
5	ICPL 20094	0.0	3.3	0.0	11.0	3.0	11.0	13.8	3.2	0.7	10.4	ghij
6	ICPL 20096	0.0	0.0	0.0	4.5	3.0	7.1	5.5	3.9	1.3	7.2	bc
7	ICPL 20097	1.3	6.8	0.0	10.6	3.1	16.4	12.6	6.0	0.0	10.7	hijk
8	ICPL 20098	3.0	0.0	0.0	9.5	4.5	48.0	1.7	3.3	3.6	11.6	klm
9	ICPL 20099	0.0	0.0	0.0	10.3	3.6	16.1	10.5	0.0	0.8	8.2	cd
10	ICPL 20100	0.9	5.7	0.0	7.0	4.9	19.4	26.9	4.2	0.0	12.3	m
11	ICPL 20101	5.6	1.9	6.8	15.3	3.5	25.1	20.3	1.2	2.3	14.1	n
12	ICPL 20102	3.5	2.1	0.0	7.0	3.3	8.5	6.9	4.1	1.7	10.1	gh
13	ICPL 20103	2.1	1.6	0.0	16.4	3.1	23.5	5.6	3.7	2.7	11.9	lm
14	ICPL 20106	0.0	2.1	0.0	13.3	4.0	9.3	1.7	3.2	1.3	8.5	de
15	ICPL 20107	0.0	0.0	3.7	10.0	3.5	21.3	23.8	2.3	1.1	11.3	jklm
16	ICPL 20109	1.3	0.0	0.0	4.8	3.0	6.4	0.0	3.9	3.6	6.0	a
17	ICPL 20110	1.2	2.3	34.8	7.3	3.5	6.4	12.1	3.0	0.0	12.4	m
18	ICPL 20113	0.0	7.8	1.0	2.5	4.7	43.1	3.6	6.8	0.0	11.2	ijkl
19	ICPL 20114	0.0	5.6	0.0	4.5	2.9	48.1	10.8	12.4	3.2	14.0	n
20	ICPL 20115	2.4	0.0	0.0	9.8	3.9	8.2	7.1	2.6	1.9	9.4	efg
21	ICPL 20116	0.0	0.0	0.0	7.0	3.5	13.0	13.0	1.6	0.0	8.1	cd
22	ICPL 20120	1.7	1.7	4.2	12.9	3.9	14.0	11.6	2.9	0.0	10.7	hijk
23	ICPL 20126	0.8	0.0	2.1	4.8	5.4	13.0	12.2	4.5	0.0	8.8	def
24	ICPL 20128	0.0	2.9	6.6	10.0	2.7	35.5	7.9	3.0	5.2	13.5	n
25	ICPL 20129	1.6	4.9	0.0	0.0	2.5	18.0	1.2	4.1	0.0	7.0	ab
26	ICPL 20132	0.0	2.6	2.6	10.4	3.0	18.6	5.0	3.1	0.0	9.9	fgh
27	ICPL 20134	0.0	0.0	3.8	7.6	6.4	41.0	2.8	3.3	0.0	10.2	ghi
28	KPBR 80-2-4	0.0	1.3	0.0	2.3	5.4	54.2	8.7	2.4	0.0	10.3	ghij
29	ICP 2376^b^	58.3	83.8	61.3	64.8	60.8	60.0	44.8	77.5	49.0	53.1	p
30	Local wilt sus. check	62.0	100.0	83.8	80.0	91.1	97.4	51.7	100.0	82.1	83.1	
	Mean	3.2	5.5	7.6	10.7	5.9	27.2	11.8	6.7	3.6		

a Mean value calculated by Bonferonni multiple comparison corrected test; BMC – Bonferonni multiple comparison;

b *Susceptible check*.

### Stability of genotype across environments

**Figure 8:**
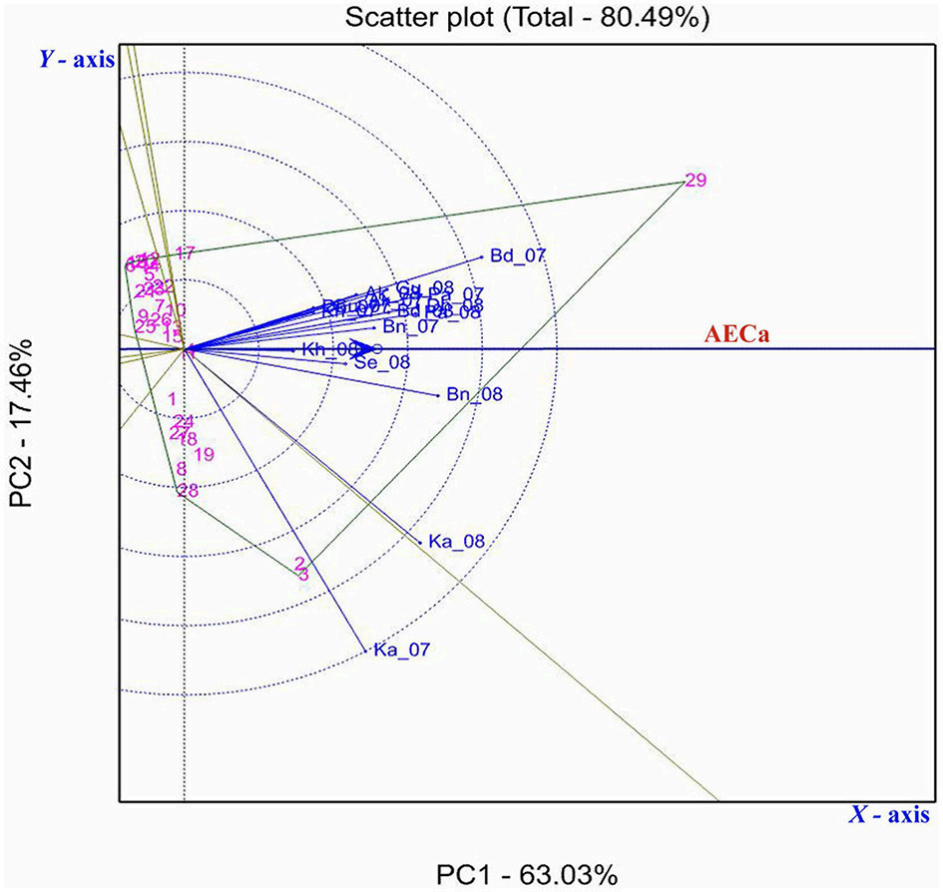
**GGE biplot showing the relationship among 18 environments based on Fusarium wilt incidence of 29 pigeonpea genotypes evaluated across nine locations in India**. First and second principal components PC1 (wilt incidence) and PC2 (resistance stability) explained 63.03 and 17.46% of total variation. The environments are denoted by first two letters of the location followed by year (2007, 07; 2008, 08); vectors are as solid blue lines. Those genotypes contributing the most to the interaction delimit the vertices of a polygon comprising the rest of accessions. A perpendicular line was drawn to each side of the polygon, forming eight individual sectors.

The GGE biplot analysis explained 80.49% of the variation (PC1 accounted for 63.03% and PC2 accounted 17.46%; Figure 8). GGE analysis showed that environments Ka-07, Ka-08, Bd-07, and Bn-08 had longer vectors than other environments indicating that they were the environments that discriminated genetic variability of the genotypes. However, Kh-08, Kh-07, Dh-07, and Gu-07 had smaller vectors indicating they were less discriminative of genotypes.

Seven out of 29 genotypes located farthest from the origin formed a seven sided polygon (Figure 8). Genotypes located at the vertices of the polygon contributed the most to the interaction, i.e., those with the highest or lowest wilt incidence. Genotype 29 (ICP 2376) was the most susceptible in all the environments except in Ka-07, where genotypes 3 (ICP 14819) and 2 (ICP 12749) were found more susceptible. The genotype 4 (ICPL 20093) was located within the polygon and nearer to the plot origin and hence was less responsive than the vertex genotypes.

AEC was created to conduct test-environment evaluation and stability of the genotype. The circles in Figure 8 represent coordinates equal to the average coordinates of the 18 marker points for environments. The blue axis passed through the origin of the biplot and in the direction of the AEC, labeled the AEC absicca (AECa), and an arrow on the AECa pointed in the direction of high wilt incidence. Twenty four genotypes at the left side of the Y-axis had stable resistance across locations. However, genotypes toward right side of the AEC ordinate had the higher wilt incidence.

Genotypes ICPL 20109 (16) and ICPL 20096 (6) had the lowest wilt incidence and placed far from the origin to left side (2.6 and 2.8% respectively) with high stability across the locations. In addition, 5 genotypes [ICPL 20115 (20), ICPL 20116 (21), ICPL 20102 (12), ICPL 20106 (14), and ICPL 20094 (5)] had the lowest level of wilt incidence with high to moderate stability across the locations. The susceptible check ICP 2376 (29) was consistently the most susceptible as seen by its placement farthest to the right of the origin of the biplot. The box plot (Figure 5) also indicated that genotypes 16 (ICPL 20109), 12 (ICPL 20102), 6 (ICPL 20096), and 20 (ICPL 20115) were most stable for their resistance against wilt, with an incidence range of 0–13% in all the environments in both the years of testing, however genotypes 3 (ICP 14819), 2 (ICP 12749), and 29 (ICP 2376) were found less stable and exhibited a high range of wilt incidence across environments.

## Discussion

The lowest wilt incidence and high stability of resistance under diverse climatic conditions are essential for the establishment of a crop and sustainable production. Our study focuses on the effect of the genetic background (genotype) and the environments impact on the *F. udum*- pigeonpea interaction. In this study, 28 genotypes with resistant reactions to wilt at a site in Patancheru were evaluated at different locations to mimic their resistance performance. However, the wilt incidence of the genotypes was found to be significantly (*p* < 0.05) influenced by environments, hence rejecting the null hypothesis.

ANOVA revealed the significant differences among the G, E, and G × E interactions. The occurrence of a significant G × E interaction indicated inconsistent wilt incidence of tested genotypes across locations, which may be due to the selection made in one environment performing poorly in another environment, and is attributed to distinct agro-ecologies with different longitude, latitude and elevations. Further it reveals that the contribution of genotypic variance for disease resistance was more than the G × E interaction indicating that most of the variation for reactions to disease was genetic. Similar results were found by other studies; Beyene et al. (2012) indicated that in a maize foliar disease resistance study the genotypic variance contribute the maximum when compared to the G × E variance. Sharma et al. (2012b) also reported that the largest portion of variability for chickpea wilt incidence was accounted by genotypes (54.4%), followed by G × E (36.7%), and E (8.9%).

The G × E interaction had a significant effect on the performance of the genotypes in specific environments, indicating that their disease reaction was impaired by interaction with the environments. For example, ICP 12749 (2) and ICP 14819 (3) expressed resistance in Akola, Badnapur, Patancheru, and Sehore but susceptibility in Bangalore, Kanpur, Khargoan and hence were not stable across environments. This variation may be attributed to the different climatic conditions, presence of different fungal variants and virulence of the pathogen at those locations. ICPL 20109 (16) and ICPL 20096 (6) expressed consistent resistant reactions in all the 18 environments and thus were highly stable. A boxplot of genotypes also confirmed the stable performance of these two genotypes as indicated by a disease score close to zero as compared with other genotypes. Further, these genotypes were placed farthest toward the left side, indicating the lowest wilt incidence across all the environments in the GGE biplot.

Projections of the environments with respect to their vector length and positive PC1 score in GGE biplots indicated high disease incidence and good levels of discriminative ability (Yan, 2001; Egesi et al., 2009). For instance, in our study Ka-07 and Ka-08 with higher vector lengths and high PC1 score supported higher wilt expression and discrimination than others. A boxplot of the environment also confirmed that the isolate from Kanpur is more virulent than the remaining isolates, indicated by a larger box size. This variation in the wilt incidence at all locations and in all genotypes may be due to the virulence of the pathogen population or difference of the genotypic characters or of the ecological conditions or combination of all the factors. Relationships between environments were specified by the hierarchical clustering of environments. Environments where disease incidence of the genotypes was reduced were grouped together in cluster I and environments where genotypes were found with higher disease incidences were grouped separately in cluster II and III. These results are in accordance with the Spearman's correlation matrix as indicated by the positive correlation of the environments within the cluster.

In conclusion, this study sheds light on the G × E interaction influencing wilt incidence in pigeonpea and calls for future studies to understand how G × E interactions influences wilt incidence. A GGE biplot, in integration with Boxplot and multiple comparison tests enabled us to identify stable genotypes to wilt (ICPL 20109, ICPL 20096, ICPL 20115, ICPL 20116, ICPL 20102, ICPL 20106, and ICPL 20094) based on their performance across diverse environments. These genotypes can be deployed in future location-specific pigeonpea resistance breeding programs.

## Author contributions

MS conceived and planned the work with significant inputs from RG and RT. RG and RT executed the experiment, collected, and compiled the data. AR and RT analyzed the data. MDS, DM, DS, and YJ contributed in the multi-location trials including data collection at their respective locations. RG and RT drafted the manuscript. MS finally edited the manuscript. All authors read the manuscript and agree with its content.

## Funding

The funding support by Department of Science and Technology (DST), Climate Change Division, Government of India, and CGIAR- Research Program Grain Legumes is acknowledged.

### Conflict of interest statement

The authors declare that the research was conducted in the absence of any commercial or financial relationships that could be construed as a potential conflict of interest.
