# Supplementary material for: Environmental Influences on Pigeonpea-Fusarium udum Interactions and Stability of Genotypes to Fusarium Wilt
Source: Front Plant Sci. 2016 Mar 7;7:253. doi: 10.3389/fpls.2016.00253 (PMC4779891; doi:10.3389/fpls.2016.00253)
Supplement: Supplementary file 1 [file Table1.docx]

**Supplementary table 1. Spearman’s Correlation coefficient between 18 environments for wilt incidence.**

| **Env** | **Ak_07** | **Ak_08** | **Bd_07** | **Bd_08** | **Bn_07** | **Bn_08** | **Dh_07** | **Dh_08** | **Gu_07** | **Gu_08** | **Ka_07** | **Ka_08** | **Kh_07** | **Kh_08** | **Pa_07** | **Pa_08** | **Se_07** | **Se_08** |
| --- | --- | --- | --- | --- | --- | --- | --- | --- | --- | --- | --- | --- | --- | --- | --- | --- | --- | --- |
| **Ak_07** | - |  |  |  |  |  |  |  |  |  |  |  |  |  |  |  |  |  |
| **Ak_08** | 0.451^*^ | - |  |  |  |  |  |  |  |  |  |  |  |  |  |  |  |  |
| **Bd_07** | 0.315^*^ | 0.326^*^ | - |  |  |  |  |  |  |  |  |  |  |  |  |  |  |  |
| **Bd_08** | 0.087 ^ns^ | 0.187 ^ns^ | 0.170 ^ns^ | - |  |  |  |  |  |  |  |  |  |  |  |  |  |  |
| **Bn_07** | 0.268^*^ | 0.307^*^ | 0.317^*^ | 0.497^*^ | - |  |  |  |  |  |  |  |  |  |  |  |  |  |
| **Bn_08** | 0.338^*^ | 0.232 ^ns^ | 0.355^*^ | 0.194 ^ns^ | 0.499^*^ | - |  |  |  |  |  |  |  |  |  |  |  |  |
| **Dh_07** | 0.285^*^ | 0.117 ^ns^ | 0.151 ^ns^ | 0.066 ^ns^ | 0.230 ^ns^ | -0.029 ^ns^ | - |  |  |  |  |  |  |  |  |  |  |  |
| **Dh_08** | 0.279^*^ | 0.379^*^ | 0.540^*^ | 0.087 ^ns^ | 0.405^*^ | 0.525^*^ | 0.244 ^ns^ | - |  |  |  |  |  |  |  |  |  |  |
| **Gu_07** | 0.033 ^ns^ | -0.041 ^ns^ | -0.048 ^ns^ | 0.097 ^ns^ | 0.281^*^ | 0.196 ^ns^ | 0.171 ^ns^ | 0.031 ^ns^ | - |  |  |  |  |  |  |  |  |  |
| **Gu_08** | 0.571^*^ | 0.259^*^ | 0.141 ^ns^ | 0.088 ^ns^ | 0.224 ^ns^ | 0.376^*^ | 0.264^*^ | 0.239 ^ns^ | 0.399^*^ | - |  |  |  |  |  |  |  |  |
| **Ka_07** | 0.203 ^ns^ | -0.018 ^ns^ | 0.228 ^ns^ | 0.407^*^ | 0.307^*^ | 0.397^*^ | 0.191 ^ns^ | -0.039 ^ns^ | 0.135 ^ns^ | 0.286^*^ | - |  |  |  |  |  |  |  |
| **Ka_08** | 0.311^*^ | 0.002 ^ns^ | 0.335^*^ | 0.367^*^ | 0.063 ^ns^ | 0.290^*^ | 0.083 ^ns^ | 0.114 ^ns^ | 0.152 ^ns^ | 0.354^*^ | 0.698^*^ | - |  |  |  |  |  |  |
| **Kh_07** | 0.352^*^ | 0.106 ^ns^ | 0.263^*^ | -0.032 ^ns^ | 0.268^*^ | 0.308^*^ | -0.053 ^ns^ | 0.159 ^ns^ | 0.156 ^ns^ | 0.126 ^ns^ | 0.002 ^ns^ | -0.021 ^ns^ | - |  |  |  |  |  |
| **Kh_08** | 0.185 ^ns^ | -0.011 ^ns^ | 0.280^*^ | 0.416^*^ | 0.205 ^ns^ | 0.269^*^ | 0.298^*^ | 0.298^*^ | 0.396^*^ | 0.017 ^ns^ | 0.256^*^ | 0.346^*^ | 0.237 ^ns^ | - |  |  |  |  |
| **Pa_07** | 0.009 ^ns^ | 0.425^*^ | 0.286^*^ | 0.273^*^ | 0.094 ^ns^ | 0.385^*^ | -0.212 ^ns^ | 0.233 ^ns^ | 0.022^*^ | 0.035 ^ns^ | 0.264^*^ | 0.424^*^ | -0.177 ^ns^ | -0.002 ^ns^ | - |  |  |  |
| **Pa_08** | 0.280^*^ | 0.397^*^ | 0.415^*^ | 0.756^*^ | 0.402^*^ | 0.218 ^ns^ | -0.002 ^ns^ | 0.313^*^ | 0.065 ^ns^ | 0.241 ^ns^ | 0.173 ^ns^ | 0.266^*^ | 0.162 ^ns^ | 0.251^*^ | 0.316^*^ | - |  |  |
| **Se_07** | 0.362^*^ | 0.354^*^ | 0.354^*^ | 0.341^*^ | 0.421^*^ | 0.362^*^ | 0.320^*^ | 0.319^*^ | 0.316^*^ | 0.316^*^ | 0.181 ^ns^ | 0.316^*^ | 0.297^*^ | 0.317^*^ | 0.317^*^ | 0.318^*^ | - |  |
| **Se_08** | 0.542^*^ | 0.322^*^ | 0.363^*^ | 0.152 ^ns^ | 0.381^*^ | 0.187 ^ns^ | 0.348^*^ | 0.257^*^ | -0.053 ^ns^ | 0.204 ^ns^ | 0.353^*^ | 0.274^*^ | 0.119 ^ns^ | 0.130 ^ns^ | 0.270^*^ | 0.187^ns^ | 0.325^*^ | - |

ns= non-significant at *P* = 0.05; *Significant at *P* = 0.01
